# To Correspondents

**Published:** 1860-05

**Authors:** 


TO CORRESPONDENTS.—Communications have been received from Drs. Fenner, Gordan, Klugé, Hargis, Bland, Reeve, and Lopez.

